# Smchd1 regulates a subset of autosomal genes subject to monoallelic expression in addition to being critical for X inactivation

**DOI:** 10.1186/1756-8935-6-19

**Published:** 2013-07-02

**Authors:** Arne W Mould, Zhenyi Pang, Miha Pakusch, Ian D Tonks, Mitchell Stark, Dianne Carrie, Pamela Mukhopadhyay, Annica Seidel, Jonathan J Ellis, Janine Deakin, Matthew J Wakefield, Lutz Krause, Marnie E Blewitt, Graham F Kay

**Affiliations:** 1 Queensland Institute of Medical Research, Brisbane, Queensland, Australia; 2 Current address: Sir William Dunn School of Pathology, University of Oxford, Oxford, UK; 3 Walter and Eliza Hall Institute, Melbourne, Victoria, Australia; 4 Research School of Biology, The Australian National University, Canberra ACT, Australia; 5 Department of Genetics, University of Melbourne, Melbourne, Victoria, Australia; 6 Department of Medical Biology, University of Melbourne, Melbourne, Victoria, Australia

**Keywords:** Clustered protocadherins, Genomic imprinting, Monoallelic expression, Smchd1, X inactivation

## Abstract

**Background:**

Smchd1 is an epigenetic modifier essential for X chromosome inactivation: female embryos lacking Smchd1 fail during midgestational development. Male mice are less affected by Smchd1-loss, with some (but not all) surviving to become fertile adults on the FVB/n genetic background. On other genetic backgrounds, all males lacking Smchd1 die perinatally. This suggests that, in addition to being critical for X inactivation, Smchd1 functions to control the expression of essential autosomal genes.

**Results:**

Using genome-wide microarray expression profiling and RNA-seq, we have identified additional genes that fail X inactivation in female *Smchd1* mutants and have identified autosomal genes in male mice where the normal expression pattern depends upon Smchd1. A subset of genes in the *Snrpn* imprinted gene cluster show an epigenetic signature and biallelic expression consistent with loss of imprinting in the absence of Smchd1. In addition, single nucleotide polymorphism analysis of expressed genes in the placenta shows that the *Igf2r* imprinted gene cluster is also disrupted, with *Slc22a3* showing biallelic expression in the absence of Smchd1. In both cases, the disruption was not due to loss of the differential methylation that marks the imprint control region, but affected genes remote from this primary imprint controlling element. The clustered protocadherins (*Pcdhα*, *Pcdhβ*, and *Pcdhγ*) also show altered expression levels, suggesting that their unique pattern of random combinatorial monoallelic expression might also be disrupted.

**Conclusions:**

Smchd1 has a role in the expression of several autosomal gene clusters that are subject to monoallelic expression, rather than being restricted to functioning uniquely in X inactivation. Our findings, combined with the recent report implicating heterozygous mutations of *SMCHD1* as a causal factor in the digenically inherited muscular weakness syndrome facioscapulohumeral muscular dystrophy-2, highlight the potential importance of Smchd1 in the etiology of diverse human diseases.

## Background

X inactivation is a developmentally regulated, epigenetic mechanism that results in monoallelic expression of X-linked genes in female mammals to achieve dosage equivalence between XX females and XY males [1]. Other examples of monoallelic expression include: (a) genomic imprinting where the parental origin of the alleles of specific genes or gene clusters are epigenetically marked during gametogenesis to be expressed exclusively from either the maternally or paternally inherited allele [2], (b) stochastic monoallelic expression, which includes allelic exclusion and is characteristic of multigene families (for example, genes encoding the olfactory [3] and pheromone receptors [4], interleukins [5], B and T cell receptors [6,7], and natural killer cell receptors [8]), (c) the random combinatorial and differential monoallelic expression of the clustered protocadherins [9-11], and (d) the apparently random widespread monoallelic expression of possibly hundreds of individual genes spread throughout the genome [12,13].

While the molecular mechanisms underlying the different forms of monoallelic expression have unique characteristics, they may be expected to share common elements. Some of the common epigenetic features identified to date include noncoding RNA (ncRNA) involvement [14-16], differential chromatin modification or DNA methylation [17-20], transchromosomal interactions [21-23], physical segregation of alleles in different nuclear compartments [24-26] and asynchronous replication [26-29].

Smchd1 (structural maintenance of chromosomes hinge domain containing 1) was identified as a semidominant suppressor of variegation in an N-ethyl-N-nitrosourea (ENU) mutagenesis screen for epigenetic modifiers [30]. The ENU derived mutant allele, named MommeD1, resulted from a nonsense mutation in the *Smchd1* gene that lead to dramatically reduced *Smchd1* transcript levels, probably the result of nonsense mediated mRNA decay [31]. Our breeding studies showed midgestation lethality of female but not male *Smchd1*^MommeD1/MommeD1^ embryos, suggestive of a failure of X inactivation in the female embryos. While the inactive X elect in female mutant embryos was decorated with *Xist* transcript and histone H3 trimethylated at lysine 27 (H3K27me3), many X-linked genes either failed to become inactivated or escaped X inactivation shortly after the initiation of silencing [31]. None of the genes tested showed the CpG island hypermethylation of the allele on the inactive X that is a normal characteristic of X inactivation [31]. Subsequently, it was shown that Smchd1 acts late in the epigenetic cascade driving X inactivation and that some X-linked genes undergo Smchd1-independent hypermethylation of the allele on the inactive X [32]. These findings demonstrated that Smchd1 function was critical for either the completion or maintenance, but not for the initiation, of X inactivation.

Several pieces of evidence suggest that Smchd1 has functions other than during X inactivation. The ENU mutagenesis screen in which *Smchd1* was identified relied upon the detection of altered variegated expression from a green fluorescent protein (GFP) transgene array integrated into an autosomal location [30,33]. Haploinsufficiency for *Smchd1* also modified expression of the *Agouti viable yellow* (*A*^vy^) allele of the *Agouti* gene (*A*), which shows variegated expression due to varying CpG methylation of an intracisternal A particle inserted upstream of the gene’s promoter [30]. In addition, although some *Smchd1*^MommeD1/MommeD1^ males survived as fertile adults in the FVB/n background, when backcrossed onto the C57Bl6/J background all homozygous males displayed perinatal lethality [34]. Further, an independently generated *Smchd1*-null allele (*Smchd1*^Gt(AD0165)Wtsi^), which phenocopies the female *Smchd1*^MommeD1/MommeD1^ phenotype, also displays perinatal lethality of males on a mixed 129/C57 genetic background [31]. We hypothesized that Smchd1 does not function solely in X inactivation but probably also epigenetically modulates expression of autosomal genes in both males and females. In this study, we have used genome-wide approaches to identify autosomal genes that have deregulated expression in the absence of Smchd1 function.

## Results

### Genome-wide expression profiling of *Smchd1* mutant embryos

**Figure 1:**
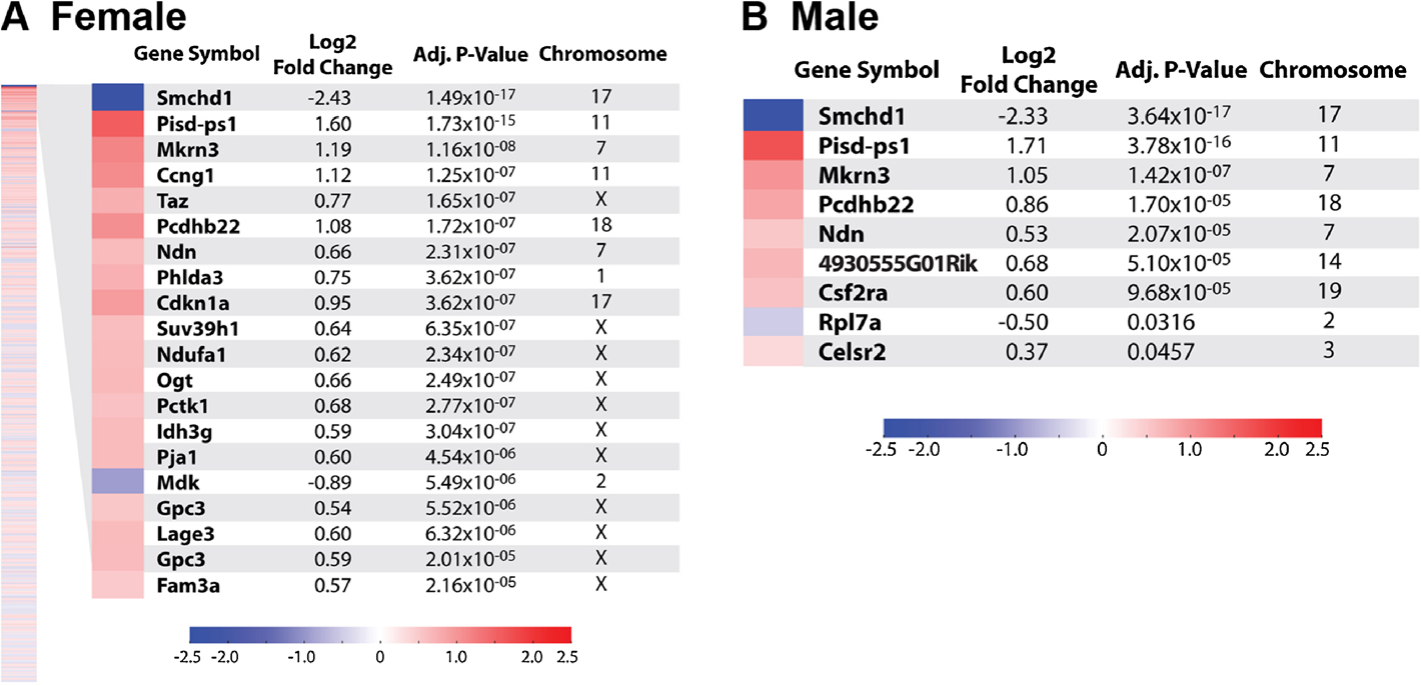
**Differential expression in *Smchd1***^**MommeD1/MommeD1 **^**versus *Smchd1***^**+/+ **^**embryos at E9.5.** Heat maps for differentially expressed genes identified in the microarray analysis comparing the expression of **(A)** female and **(B)** male embryos. The lists of differentially expressed genes were sorted in order of level of significance with multiple testing correction and those showing a significant difference between the genotypes (adjusted *P* < 0.05) are displayed on the heat maps. For the female comparison, the top 20 genes are shown, while for the male comparison all genes that were significantly different are shown.

We undertook genome-wide microarray expression profiling to screen for differences between *Smchd1*^MommeD1/MommeD1^ and *Smchd1*^+/+^, male and female embryos (*n* = 4 for each genotype and sex combination) at E9.5 (Figure 1, Additional files 1 and 2). Males and females were analyzed separately because the females are likely to have deregulated expression of autosomal genes as a secondary consequence of X inactivation failure in addition to autosomal genes whose expression is directly regulated by Smchd1. As expected, because the analysis involved comparison of *Smchd1*^MommeD1/MommeD1^ versus *Smchd1*^+/+^, the most significantly decreased transcript levels in all comparisons was *Smchd1*.

After filtering out the genes represented by poor quality probes on the microarray, as determined by the Illumina BeadChip Probe Reannotation datasets [35], and counting genes represented by multiple probes only once, the list of unique genes showing significantly altered transcript levels in female *Smchd1*^MommeD1/MommeD1^ embryos (356 genes, adjusted *P* < 0.05, Figure 1A, Additional file 1) was much more extensive than that for males (9 genes, adjusted *P* < 0.05, Figure 1B, Additional file 2). The differentially expressed genes in females included 66 X-linked genes with increased transcript levels and no X-linked genes with decreased levels. Autosomal differentially expressed genes in females (290 genes in total) were distributed between those with increased transcript levels (157 genes) and those with decreased levels (133 genes) in *Smchd1*^MommeD1/MommeD1^ compared with *Smchd1*^+/+^ embryos.

The cell cycle related genes *Ccng1* and *Cdkn1a* were high in the ranked list of significantly upregulated transcripts in female mutant embryos but not in male mutants. A likely explanation for the altered expression levels of these genes is that the female *Smchd1*^MommeD1/MommeD1^ embryos were beginning to fail at this stage of development despite looking normal at the macroscopic level. This was supported by gene set enrichment analysis of the autosomal gene classes altered in female mutants (Additional file 3). Gene categories associated with the apoptosis pathway were significantly altered, indicating that failure of X inactivation initiates cell death at this stage in at least a subset of embryo tissues. Gene categories involved in developmental processes normally active in this stage of development and biosynthetic categories were also significantly altered, consistent with failure of embryo growth and development. These results confirmed our previous study showing midgestation lethality due to failure of X inactivation in female *Smchd1* mutants [31] and considerably extended the list of genes failing X inactivation (from 7 of 16 genes previously tested [31] to 66 genes in the current study, for a nonredundant list of 70 genes failing X inactivation). Most of the genes identified in this study were previously classified as dependent on Smchd1 function for methylation of their CpG island (48 of 66) [32], while three genes (*Ebp*, *Magee1*, and *Ndufa1*) were previously shown to have an intermediate level of methylation in *Smchd1* mutants; the remaining 15 genes were not previously classified. Importantly, no X-linked genes showed significantly altered transcript levels in the male embryo comparison. The ability to detect statistically significant differential expression depends on several factors, including the signal intensity (A value) and intersample variability. Genes with tissue-specific expression, low absolute expression, or lower quality probes on the array are likely to produce false negatives, hence some X-linked differentially expressed genes could have been missed by this analysis. We therefore examined the observed fold change of all expressed X-linked genes (336 genes) compared with all expressed autosomal genes (9065 genes), where expressed genes were defined as having an *A* value > 7.0. The mean log2 fold change of expressed X-linked genes was significantly higher than zero (mean = 0.1730, *n* = 336, Wilcoxon signed rank test *P* value < 2.2x10^−16^, Additional file 4).

**Figure 2:**
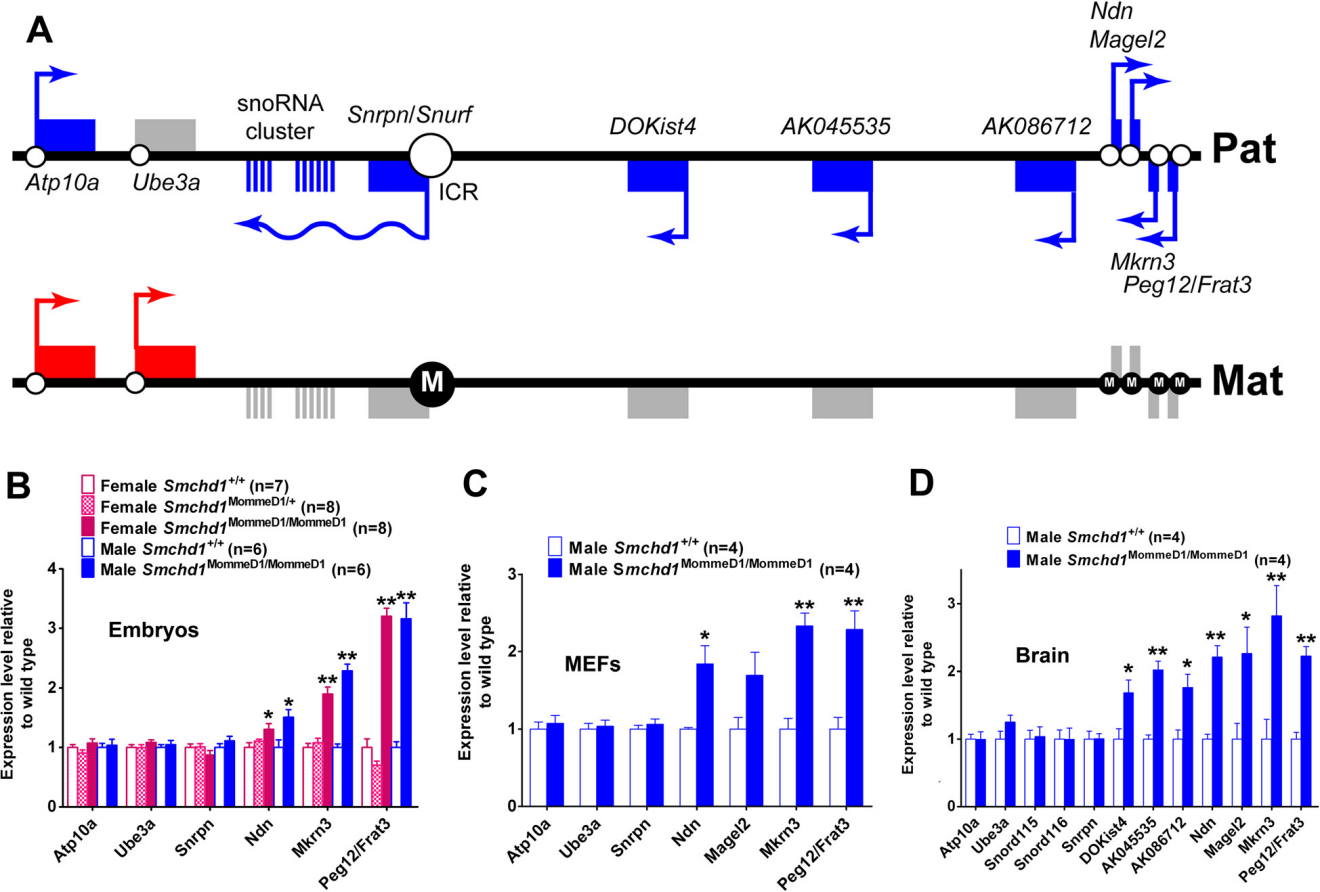
**A subset of imprinted genes in the *Snrpn *cluster shows increased expression in *Smchd1 *mutants. (A)** The *Snrpn* imprinted gene cluster, where those genes expressed from the paternal allele are shown in blue and those expressed from the maternal allele are in red. CpG islands are represented by circles on the line and where these represent an sDMR (small circles) or imprint control region (ICR) (large circle) the methylation status is indicated (M inside the filled circle for the methylated allele or unfilled circle for the unmethylated allele). Quantification of the expression levels of imprinted genes or transcripts within the *Snrpn* cluster by qRT-PCR measured using RNA derived from: **(B)** male and female E9.5 embryos, **(C)** mouse embryonic fibroblasts (MEF) derived from male E14.5 embryos, and **(D)** whole brain from adult male mice. The synthesis of first-strand cDNA from E9.5 embryo RNA was primed with oligo dT, but for the analysis of transcript levels in MEFs and brain the cDNA synthesis was primed with a cocktail of the reverse primers used for qRT-PCR. In each case the qRT-PCR signal was normalized relative to that of *Rala* and plotted relative to the corresponding *Smchd1*^+/+^ sample. The genotype, sex, and number of replicates are indicated in each case. Statistical analysis was performed using the *t* test. ** *P* < 0.01 and * *P* < 0.05 compared with wildtype. Error bars indicate standard error.

The nine genes showing significantly altered transcript levels in males were also altered to a similar degree (fold change and level of statistical significance) in females. Prominent within both male and female gene lists with increased transcript levels were *Ndn* and *Mkrn3*, two genes known to be subject to genomic imprinting, which are located in the *Snrpn* imprinted gene cluster on mouse chromosome 7 [15q11-13 in human beings] [36,37]. The *Snrpn* imprinted gene cluster contains both paternally and maternally expressed imprinted genes (Figure 2A), with mutations within the cluster being responsible for Angelman [OMIM 105830] and Prader-Willi [OMIM 176270] syndromes in human beings.

Several of the clustered protocadherin genes had an altered expression. *Pcdhβ22* was significantly differentially expressed in both males and females, with increased transcript levels in *Smchd1* mutants. *Pcdhβ3* and *Pcdhβ16* also showed significantly altered expression, but only in the female list. *Pcdhα*, *Pcdhβ*, and *Pcdhγ* are located in a cluster on mouse chromosome 18 [38] and display an unusual form of monoallelic expression involving random and combinatorial expression with each individual neuron expressing a unique combination of the α, β and γ isoforms [9,10,39]. It has been proposed that this results in enormous combinatorial diversity of the protocadherins displayed on the surface of neurons and may confer a unique identity on each neuron [39]. To confirm the results obtained from the microarray analysis we performed qRT-PCR analysis for the top four deregulated genes in males using RNA derived from E9.5 embryos (Additional file 5). All genes tested displayed significantly increased transcript levels (*P* < 0.01 for *Mkrn3*, *Pisd*-*ps1*, and *Pcdhβ22*; *P* < 0.05 for *Ndn*) in both male and female *Smchd1*^MommeD1/MommeD1^ mutant embryos compared with *Smchd1*^+/+^ embryos.

Given the phenotypic variability displayed by male *Smchd1* mutants on different genetic backgrounds, we subjected an independent set of male E9.5 embryos of a different genetic background to RNA-seq analysis (*n* = 3 for each genotype, *Smchd1*^+/+^ and *Smchd1*^MommeD1/MommeD1^). These embryos were generated from mice where the *Smchd1*^MommeD1^ mutation had been backcrossed to 129 T2/SvEms background for four generations. A partially overlapping, but still relatively short list of 24 genes showed significant differences in gene expression between the genotypes (Additional file 6). It is likely that the variation in gene expression differences detected using the microarray and RNA-seq analysis results from a combination of the different genetic background used in each study and the inherent sensitivity and selectivity differences of the two methods used to assay gene expression. The differentially expressed genes detected by both analyses again pointed to disruption of the *Snrpn* imprinted gene cluster, with *Ndn* (adjusted *P* = 0.0059) and *Peg12*/*Frat3* (adjusted *P* = 0.0018) being significantly differently expressed approximately 2-fold in the RNA-seq analysis. The other gene detected by both analyses was *Pisd*-*ps1*, about which little is known.

What was evident in the RNA-seq analysis, and could not have been detected by the microarray, was a novel gene that flanked and spliced across *Ndn*, *Magel2*, and *Mkrn3* (Additional file 7). This novel gene corresponded to [AK142799 Genbank], a 3478 bp mRNA identified in a RIKEN *Mus musculus* 15-day embryo head cDNA library. The mRNA encodes a hypothetical tyrosine-rich region profile/EGF-like domain-containing protein. Expression levels of this gene appeared to be increased in *Smchd1*^MommeD1/MommeD1^ compared with *Smchd1*^+/+^ embryos (although not to statistically significant levels, owing to relatively low sequence read count), similar to the imprinted genes *Ndn*, *Magel2* and *Mkrn3*, which lie within its intron.

### Specific analysis of the *Snrpn* cluster of imprinted genes

Since some genes within the *Snrpn* imprinted cluster showed statistically significant increased transcript levels in the absence of Smchd1 in both the microarray and the RNA-seq analyses, we used qRT-PCR to analyze further genes specifically from the *Snrpn* imprinted gene cluster in E9.5 embryos (Figure 2B). We found that *Ndn*, *Mkrn3* and *Peg12*/*Frat3* were significantly deregulated but *Snrpn*, *Ube3a and Atp10a* were not. *Snrpn* normally displays imprinted expression in all tissues [40], while *Ube3a* has imprinted expression only in the brain [41-43]. There are conflicting reports regarding the imprinted status of *Atp10a* in both human beings [44-46] and mice [47-49]. The inclusion of *Smchd1*^MommeD1/+^ female embryos in this analysis demonstrated that haploinsufficiency for *Smchd1* was not sufficient to cause detectable disruption of expression for the affected genes. In addition, *Magel2* transcript levels were not sufficient to allow reliable detection.

Similar results were obtained for *Ndn*, *Mkrn3*, *Peg12*/*Frat3*, *Snrpn*, *Ube3a*, and *Atp10a* in mouse embryonic fibroblast (MEF) cultures (Figure 2C) as for E9.5 embryo samples. *Magel2* was more reliably detectable in MEFs but, while its transcript levels appeared to be increased in *Smchd1*^MommeD1/MommeD1^ MEFs, the difference was not statistically significant (*P* = 0.081012) for the four independently derived MEF cultures used.

While most genes within the *Snrpn* cluster have their highest expression levels in the brain (for example, *Peg12*/*Frat3*[50,51]), some are only expressed in the brain (for example, *snoRNAs*[52,53]) or are only expressed in an imprinted manner in the brain (for example, *Ube3a*[43]). In addition, a recent study [54] has identified transcripts uniquely expressed in the brain that originate from imprinted loci located between *Snrpn* and *Ndn*. These transcripts encode either ncRNAs or predicted miRNAs, and are normally expressed from only the paternal allele. Analysis using RNA isolated from the brains of adult *Smchd1*^+/+^ and *Smchd1*^MommeD1/MommeD1^ males (Figure 2D) also revealed similar results for *Ndn*, *Mkrn3*, *Magel2*, and *Peg12*/*Frat3*, with significantly increased transcript levels in the absence of Smchd1. A subset of the recently identified imprinted genes spread across the region between *Ndn* and *Snrpn* (that is, *DOKist4*, *AK045535*, and *AK086712*) were also tested and found to be significantly increased. The imprinted *snoRNAs* (*SnoRD115* and *SnoRD116*) that lie between *Snrpn* and *Ube3a* were expressed equally in *Smchd1*^+/+^ and *Smchd1*^MommeD1/MommeD1^ brain.

### Epigenetic signature and allelic expression of disrupted imprinted genes

We considered that the increased transcript levels seen in *Smchd1* mutants (for example, *Ndn*, *Magel2*, *Mkrn3*, *Peg12*/*Frat3*, and the ncRNA transcripts originating from loci between *Snrpn* and *Ndn*) could result from loss of imprinting and the biallelic expression for these genes rather than an increased level of monoallelic expression. Imprinted genes can be identified by their overlapping permissive and nonpermissive epigenetic markings, where the expressed allele is marked by H3 dimethylated at lysine 4 (H3K4me2) and the nonexpressed allele by CpG methylation [55], or alternatively H3 trimethylated at lysine 4 (H3K4me3) and H3 trimethylated at lysine 9 (H3K9me3) [56], respectively. Thus, loss of imprinting and biallelic expression would result in easily detectable changes of these epigenetic signatures with increased levels of the permissive marks (that is, H3K4me2 or H3K4me3) and reduced levels of the nonpermissive marks (that is, CpG methylation or H3K9me3).

To analyze DNA methylation, we used methylated DNA immunoprecipitation (MeDIP) followed by qPCR to quantify the degree of enrichment. Differential methylation of the parental alleles of the imprint control region (ICR), associated with *Snrpn*[57], is the primary mechanism that directs imprinted expression within the cluster [58,59]. It is the maternally inherited allele of the *Snrpn* ICR that is methylated, while the paternally inherited allele is not methylated (Figure 2A). A secondary (or somatic) differentially methylated region (sDMR) is associated with the CpG island of *Ndn*, *Mkrn3*, *Magel2*, and *Peg12*/*Frat3*[51,60-62]. The sDMR becomes methylated on the maternally inherited allele at postzygotic stages, usually postimplantation, rather than being established in the gametes.

**Figure 3:**
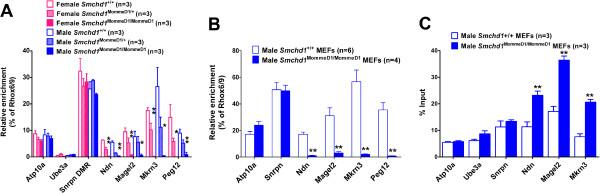
**The epigenetic signature of deregulated genes in the *Snrpn *cluster is consistent with biallelic expression.** The relative level of DNA methylation was quantified by qPCR of MeDIP recovered DNA in samples from **(A)** male and female E9.5 embryos, and **(B)** MEFs isolated from male E14.5 embryos. **(C)** The relative level of H3K4me2 for each gene was quantified by qPCR of ChIP recovered material in chromatin derived from MEFs isolated from male E14.5 embryos. The qPCR signal was normalized relative to that of *Rhox6*/*9* for MeDIP or relative to input for the H3K4me2 ChIP. Results are plotted relative to the corresponding *Smchd1*^+/+^ sample. The genotype, sex, and number of replicates are indicated in each case. Statistical analysis was performed using the *t* test. In **(A)** **P < 0.01 or *P < 0.05 for *Smchd1*^MommeD1/MommeD1^ compared with *Smchd1*^+/+^. Statistical significance not shown for the comparison of *Smchd1*^+/+^ compared with *Smchd1*^MommeD1/+^. In **(B)** and **(C)** ** *P* < 0.01 compared with wildtype. Error bars indicate standard error. ChIP, chromatin immunoprecipitation.

MeDIP analysis of DNA from E9.5 *Smchd1*^+/+^, *Smchd1*^MommeD1/+^, and *Smchd1*^MommeD1/MommeD1^ male and female embryos (Figure 3A) showed that the *Snrpn* ICR and the CpG island of *Atp10a* had no difference in DNA methylation levels between the genotypes. Recovery of MeDIP material indicated that in *Smchd1*^MommeD1/MommeD1^ embryos the sDMRs associated with *Ndn*, *Magel2*, *Mkrn3*, and *Peg12*/*Frat3* were almost completely unmethylated compared with *Smchd1*^+/+^ embryos. The *Smchd1*^MommeD1/+^ embryos had a level of methylation intermediate between *Smchd1*^+/+^ and *Smchd1*^MommeD1/MommeD1^ embryos for *Ndn*, *Magel2*, *Mkrn3*, and *Peg12*/*Frat3*. This intermediate methylation level (Figure 3A) did not translate into a detectable effect on the expression of these genes (Figure 2A). This result was similar to that previously shown in *Smchd1*^MommeD1/+^ female embryos, where expression of many X-linked genes and X inactivation of an X-linked GFP transgene was similar to *Smchd1*^+/+^ embryos despite X-linked genes showing a level of CpG island methylation intermediate between *Smchd1*^+/+^ and *Smchd1*^MommeD1/MommeD1^ embryos [31]. While *Ube3a* displays imprinted expression in the brain, its CpG island is not strongly differentially methylated and remains unmethylated on both alleles [63]. The MeDIP result for *Ube2a* is consistent with this.

These results indicated that in the absence of Smchd1 the levels of methylation of the *Snrpn* ICR were retained, but the sDMRs associated with *Ndn*, *Magel2*, *Mkrn3*, and *Peg12*/*Frat3* were unmethylated. Similar results were obtained from MeDIP of MEF DNA (Figure 3B). The result was confirmed by bisulfite sequencing of MEF DNA (Additional file 8), which also confirmed that the differential methylation of the *Snrpn* ICR was retained in the absence of Smchd1. The imprinted loci lying between *Snrpn* and *Ndn* do not contain any annotated CpG islands in MM10 and were not analyzed.

In parallel, we used chromatin immunoprecipitation (ChIP) to analyze H3K4me2, marking the same region in MEFs. Similar ChIP in human cells shows that the CpG island of *NDN* and the Prader-Willi syndrome imprinting center (located in exon 1 of the *SNRPN* gene) carry H3K4me2 and H3K4me3 marks on the expressed paternal allele but not on the nonexpressed maternal allele [64,65]. The results (Figure 3C) showed that levels of H3K4me2 were approximately doubled for each gene with deregulated expression and not different for genes whose expression level was indistinguishable in *Smchd1*^MommeD1/MommeD1^ compared with *Smchd1*^+/+^ controls. This is consistent with loss of imprinting for *Ndn*, *Mkrn3*, and *Magel2*, and both alleles being marked with this permissive histone modification in the absence of Smchd1. H3K4me2 levels on *Peg12*/*Frat3* were not tested. No difference in H3K4me2 levels was seen at the *Snrpn* ICR, consistent with our finding that loss of Smchd1 did not affect the *Snrpn* expression.

**Figure 4:**
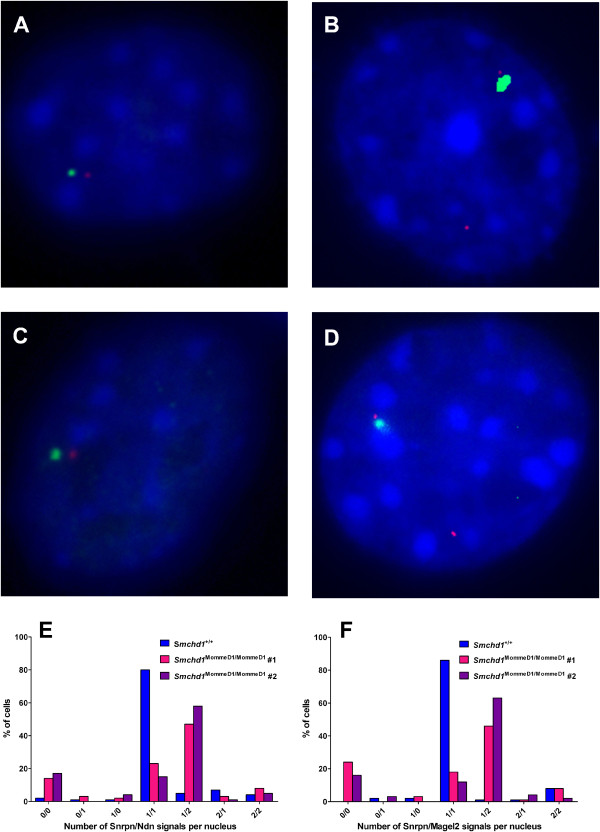
**RNA-FISH showing biallelic expression of deregulated genes in the *Snrpn *cluster.***Snrpn* (green) and *Ndn* (red) RNA-FISH signals on **(A)***Smchd1*^+/+^ and **(B)***Smchd1*^MommeD1/MommeD1^ MEFs. *Snrpn* (green) and *Magel2* (red) RNA-FISH signals on **(C)***Smchd1*^+/+^ and **(D)***Smchd1*^MommeD1/MommeD1^ MEFs. Images representative of the predominant hybridizing pattern for each genotype and gene combination are shown. The number of RNA-FISH signals per nucleus for **(E)***Snrpn*/*Ndn* and **(F)***Snrpn*/*Magel2* were counted (100 randomly selected nuclei counted in each case) and plotted for each line of MEFs (*Smchd1*^+/+^, *Smchd1*^MommeD1/MommeD1^#1, and *Smchd1*^MommeD1/MommeD1^#2). The genotypes are indicated in each case.

In combination, these changes in epigenetic signature and gene expression in *Smchd1* mutants are completely consistent with loss of imprinting resulting in biallelic expression of *Ndn*, *Magel2*, *Mkrn3*, and *Peg12*/*Frat3* but with imprinting being maintained at the other imprinted genes within the cluster (that is, *Snrpn* and *Ube3a*). From our gene expression analysis *DOKist4*, *AK045535*, and *AK086712* are also likely to be subject to loss of imprinting but not the imprinted *snoRNAs* (that is, *SnoRD115* and *SnoRD116*). To definitively demonstrate the allelic expression of genes within the *Snrpn* imprinted gene cluster we used RNA-FISH on MEFs derived from E14.5 *Smchd1*^+/+^ compared with *Smchd1*^MommeD1/MommeD1^ embryos. We compared the number of signals in the nucleus resulting from FISH probes that detect *Snrpn* and either *Ndn* or *Magel2* transcript (Figure 4A-D). In *Smchd1*^+/+^ MEFs *Snrpn*, *Ndn*, and *Magel2* were expressed monoallelically in approximately 80% of nuclei (Figure 4E and F). In *Smchd1*^MommeD1/MommeD1^ MEFs, monoallelic expression of these genes was only seen in 12 to 23% of nuclei, with between 46% and 63% of nuclei showing biallelic expression of *Ndn* or *Magel2* but monoallelic expression of *Snrpn*.

### Identification of other imprinted genes showing loss of imprinting in *Smchd1* mutants

Lack of DNA methylation at the sDMR associated with *Ndn*, *Magel2*, *Mkrn3*, and *Peg12* was the most obvious phenotype associated with homozygous mutation of *Smchd1*. The level of CpG methylation of the ICR associated with the *Snrpn* cluster showed no difference between *Smchd1*^MommeD1/MommeD1^ and *Smchd1*^+/+^ samples. We undertook qPCR MeDIP analysis of the ICR or sDMR associated with other imprinted genes using embryo- and male-MEF-derived DNA. In these analyses, only the sDMR associated with the *Cdkn1c* gene showed a significant decrease in CpG methylation levels in *Smchd1*^Mommed1/MommeD1^ samples; however, this was only observed in embryo-derived DNA (Additional file 9A), not in the DNA derived from MEFs (Additional file 9B). The degree of DNA methylation loss at *Cdkn1c* in the *Smchd1*^Mommed1/MommeD1^ samples was not as marked as for the sDMR associated with *Ndn*, *Magel2*, *Mkrn3*, and *Frat3*/*Peg12*. We were not able to demonstrate any significant loss of methylation at the ICR of any imprinted gene cluster that we tested.

The use of single nucleotide polymorphisms (SNPs) to determine the parental allele of origin of expressed alleles is the most widely accepted method for determining whether genes are subject to (or failing) genomic imprinting. We had previously backcrossed the *Smchd1*^MommeD1^ mutation from the FVB/n background (in which the ENU mutagenesis screen that identified *Smchd1* was undertaken) onto the C57Bl6/J background for more than ten generations. While none of the genes in the *Snrpn* imprinted gene cluster carries SNPs between these genetic backgrounds, several other imprinted genes do. This is particularly the case for imprinted genes that are expressed in an imprinted manner uniquely in the placenta. To identify more imprinted genes with loss of imprinting in *Smchd1* mutants, we analyzed the placentas of F1 (C57Bl6/J × FVB/n) E14.5 male embryos (*n* = 5 for each genotype, *Smchd1*^+/+^ and *Smchd1*^MommeD1/MommeD1^). To ensure that we were able to dissect out the embryonic portion of the placenta free from contamination by maternal tissue, we also included a ubiquitously expressed GFP transgene (UBI-GFP) [66] in the sires. The genotypes of the mice set up in the cross were as follows: (C57Bl6/J; *Smchd1*^MommeD1/+^, UBI-GFP^T/+^) sire crossed to (FVB/n; *Smchd1*^MommeD1/+^) dam. Only the brightly GFP fluorescent embryonic portion of the placenta was used for RNA-seq analysis. Very few genes showed differential levels of expression between the genotypes (Additional file 10), with only four RefSeq genes reaching statistical significance *Smchd1*, *Mmp12*, *Cyp1a1*, and *Apoa1* (adjusted *P* < 0.05).

**Figure 5:**
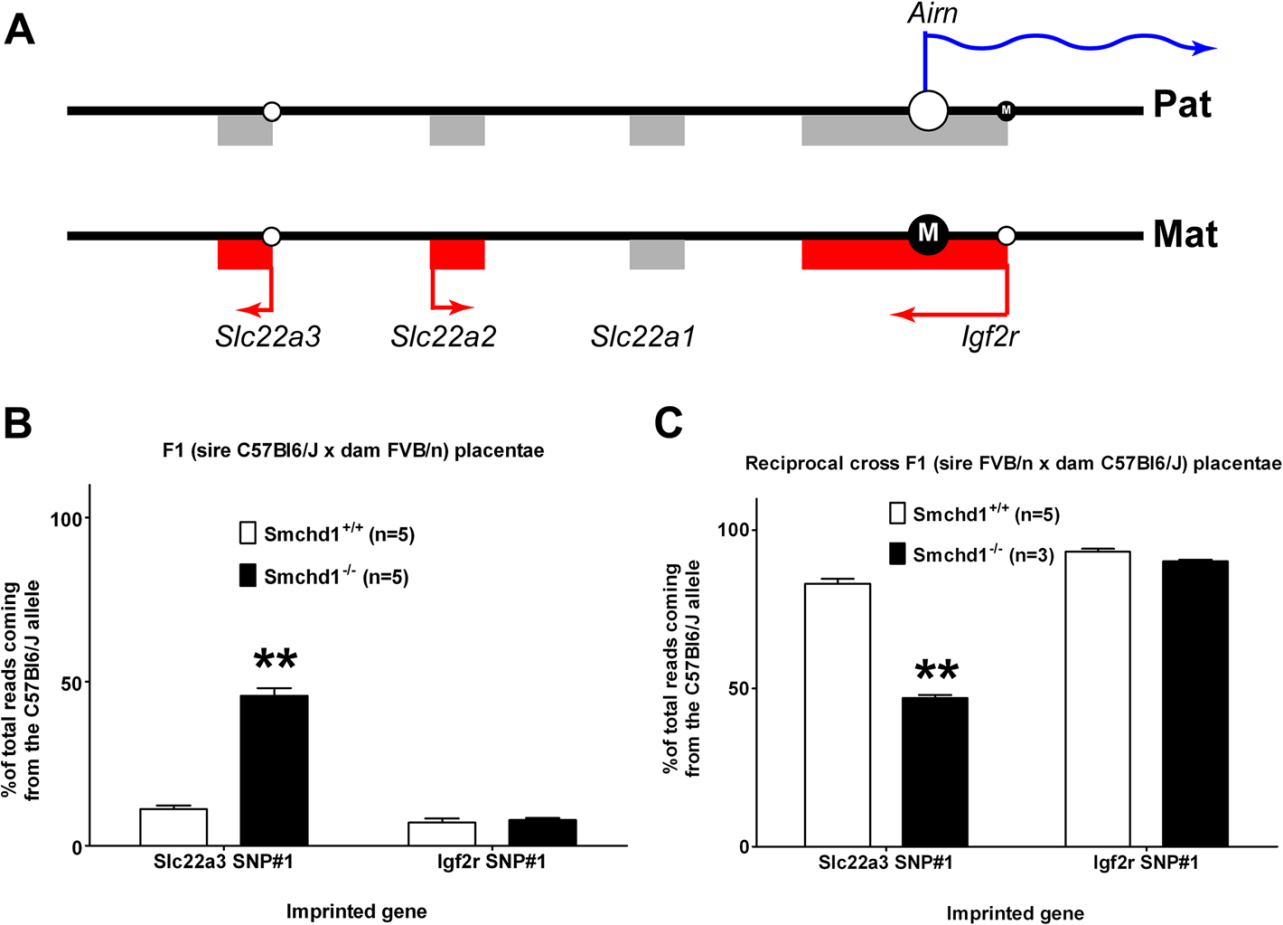
**Loss of imprinting at the *Igf2r *imprinted gene cluster in placenta in the absence of *Smchd1*. (A)** The *Igf2r* imprinted gene cluster shows imprinting in the placenta, where genes expressed from the paternal allele are shown in blue and those expressed from the maternal allele are in red. CpG islands are represented by circles on the line and where these represent an ICR (large circle) the methylation status is indicated (M inside the filled circle for the methylated allele or unfilled circle for the unmethylated allele). **(B)** In *Smchd1*^+/+^ F1 (C57Bl6/J sire × FVB/n dam) placental expression of both *Igf2r* and *Slc22a3* is predominantly from the maternal FVB/n allele with only a minor proportion from the paternal C57Bl6/J allele, but in *Smchd1*^MommeD1/MommeD1^ placentas, expression of *Slc22a3* becomes biallelic while expression of *Igf2r* remains imprinted with predominant expression of the maternal allele. **(C)** The same result is seen in the reciprocal cross F1 (FVB/n sire × C57Bl6/J dam) except here the maternal allele is derived from the C57Bl6/J dam. The genotype and number of replicates are indicated in each case. Statistical analysis was performed using the t test. ** *P* < 0.01 compared with wildtype. Error bars indicate standard error.

Using SNP analysis, we identified the *Igf2r* imprinted cluster as being disrupted in *Smchd1* mutants. The cluster includes the imprinted genes *Igf2r*, *Slc22a2*, and *Slc22a3*, which are preferentially expressed from the maternally inherited allele, and the *Airn* ncRNA, which is preferentially expressed from the paternally inherited allele (Figure 5A). *Igf2r* and *Airn* are imprinted in all tissues, but *Slc22a2* and *Slc22a3* are imprinted only in the placenta, with *Slc22a3* becoming biallelically expressed from E15.5 onwards [67]. In *Smchd1* mutant placenta, the allelic pattern of *Igf2r* expression is identical to that in wildtype placenta with expression predominantly from the maternal allele, but the *Slc22a3* gene showed biallelic expression rather than the predominant maternal allele shown by wildtype placenta of the same age (Figure 5B, Additional file 11). This finding was confirmed by analysis of placentas derived from a reciprocal cross: a (FVB/n; *Smchd1*^MommeD1/+^) sire crossed to a (C57Bl6/J; *Smchd1*^MommeD1/+^) dam (Figure 5C). This formally demonstrates loss of imprinting in the absence of Smchd1. No other imprinted genes that were amenable to analysis in this experiment (that is, showing imprinted expression in the placenta and carrying suitable SNPs) showed similar statistically significant loss of imprinting (Additional file 11). Bisulfite analysis confirmed that Smchd1 loss did not alter the differential methylation of the ICR that lies within exon 2 of *Igf2r* and that controls imprinting of the *Igf2r* imprinted cluster of genes (Additional file 12).

The SNP analysis also revealed exclusive expression of the FVB/n alleles of genes within a region of chromosome 17 surrounding *Smchd1* (that is, from *Tgif1* at position [Chr17:70844412 MM10] to *Ttc27* at [Chr17:74770280 MM10]. This resulted from FVB/n alleles of genes linked to the mutant *Smchd1*^MommeD1^ allele being retained in the backcross to C57Bl/6 J. While this finding was not relevant to the genomic imprinting data for the *Igf2r* imprinted cluster, it indicated that we were successful in obtaining clean embryonic placental tissue for the analysis and defined the region of the FVB/n chromosome surrounding the *Smchd1*^MommeD1^ that was retained in our backcrossed animals.

### The clustered protocadherin genes show altered expression in *Smchd1* mutants

**Figure 6:**
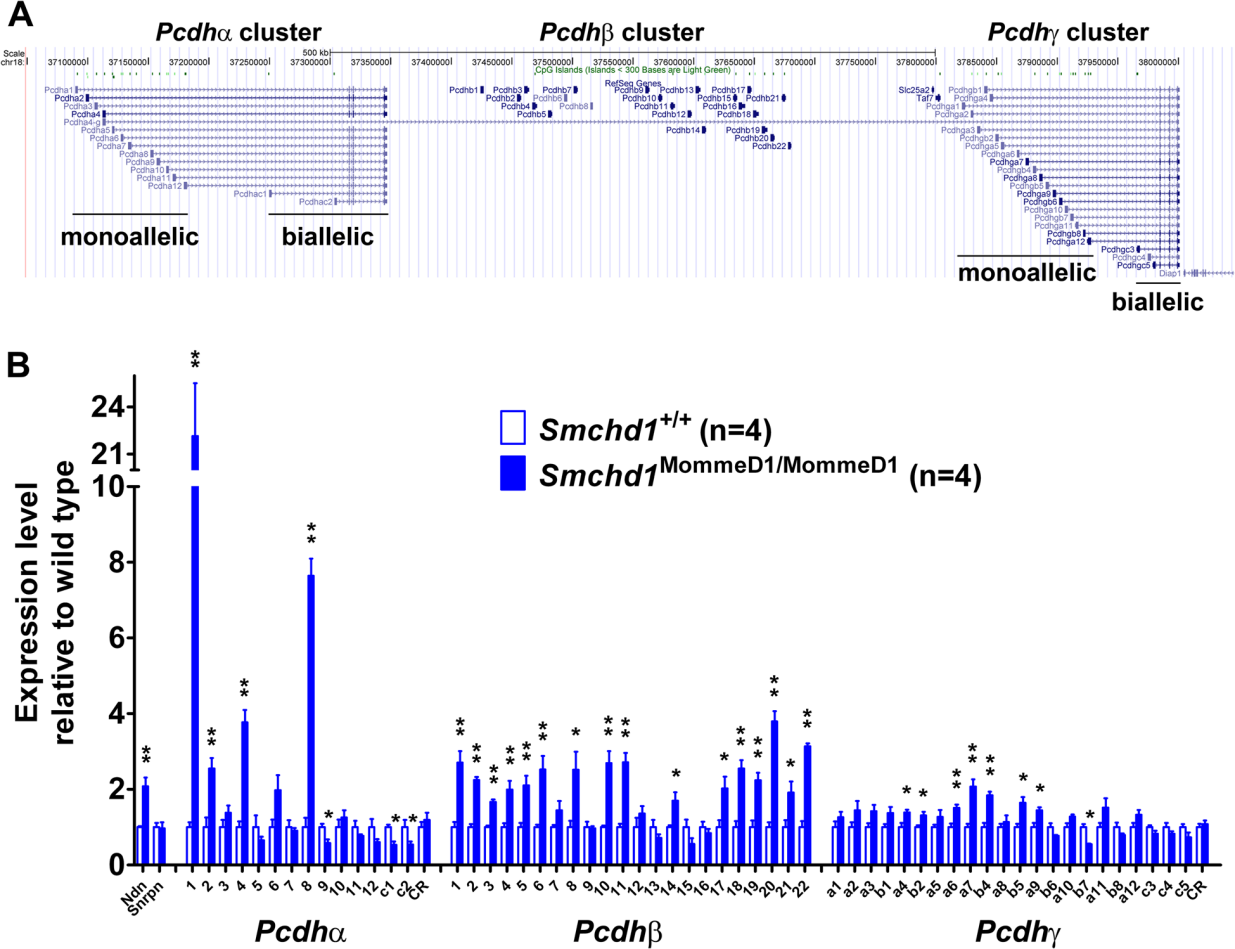
**The clustered protocadherin genes show altered expression levels in *Smchd1 *mutants. (A)** The clustered protocadherin genes (*Pcdhα*, *Pcdhβ*, and *Pcdhγ*) lie in a cluster on mouse Chr:18 and are expressed in a unique manner. Transcripts from the *Pcdhα* and *Pcdhγ* genes show alternative first exons that are monoallelically expressed in a random combinatorial manner and spliced to downstream exons that are biallelically expressed. The *Pcdhβ* genes exist as individual genes. **(B)** The expression levels of the protocadherin genes (*Pcdhα*, *Pcdhβ*, and *Pcdhγ*) were quantified by qRT-PCR using RNA derived from whole adult male mouse brains. The synthesis of first-strand cDNA was primed with a cocktail of the reverse primers used for qRT-PCR. In each case, the qRT-PCR signal was normalized relative to that of *Rala* and plotted relative to the corresponding *Smchd1*^+/+^ sample. Statistical analysis was performed using the *t* test. ** *P* < 0.01 and * *P* < 0.05 compared with wildtype. Error bars indicate standard error.

Previously, we had shown that expression of members of the clustered protocadherin family was deregulated in *Smchd1* mutant MEFs (normal and transformed) [34]. Since *Pcdhβ22* also showed significantly altered transcript levels in the microarray analysis of E9.5 embryos, we considered the possibility that Smchd1 might function in the mechanism controlling the random combinatorial monoallelic expression of the clustered protocadherins (Figure 6A). We screened the full transcript set from all three types of clustered protocadherin gene for deregulated expression levels in the adult male brain and found that all three clusters showed altered expression in *Smchd1* mutants (Figure 6B). The strongest changes occurred in the *Pcdhα* cluster, where *Pcdhα1* was increased 22-fold and *Pcdhα8* was increased approximately 7.5-fold. Three *Pcdhα* transcripts (*Pcdhα9*, *PcdhαC1*, and *PcdhαC2*) were significantly decreased. Almost all members of the *Pcdhβ* cluster (*Pcdhβ1*-*6*, *8*, *10*, *11*, *14*, *17* to *22*) displayed increased transcript levels ranging from 2 to 4-fold, while the *Pcdhγ* cluster was least affected both in terms of fold change and number of alternative transcripts displaying some alteration.

## Discussion

We initially identified *Smchd1* as an epigenetic modifier in an ENU mutagenesis screen [30] and demonstrated a critical role for Smchd1 in either the completion or maintenance, but not the initiation, of X inactivation [31]. This study considerably extends the known genes failing X inactivation in *Smchd1* mutants (7 previously published, 66 in the current study, for a nonredundant total list of 70 genes). While it is clear that some genes do not fail X inactivation in *Smchd1* mutants [32], we believe that the current set of genes does not reflect the full extent of genes failing X inactivation since it is likely that many X-linked genes would not be expressed at sufficient levels at this developmental stage to be detected.

Our main aim was to identify autosomal genes whose correct expression was directly dependent upon Smchd1 function. We had reasoned that there would be many autosomal genes in female *Smchd1* mutants that would display deregulated expression that was secondary to failure of X inactivation. To ensure that we could identify autosomal genes that were directly regulated by Smchd1, we analyzed males separately from females and concentrated on genes whose expression was altered to a similar degree in both males and females. Our analysis revealed a relatively short list of nine genes showing statistically significant altered expression in *Smchd1* mutant males; all of these genes were similarly deregulated in females.

Amongst this list were two genes (*Ndn* and *Mkrn3*) from the *Snrpn* imprinted gene cluster with increased levels of expression. Specific analysis revealed further genes from the cluster (*Magel2*, *Peg12*/*Frat3*, *DOKist4*, *AK045535*, and *AK086712*) showing increased expression in *Smchd1* mutants. The deregulated genes displayed epigenetic signatures consistent with biallelic expression and loss of imprinting. RNA-FISH showed that *Ndn* and *Magel2* displayed biallelic expression but *Snrpn* retained monoallelic expression. From this result it is clear that the *Snrpn* imprinted cluster has two distinct parts in terms of its imprinting mechanism; (a) the region distal to the *Snrpn* start site containing genes, whose imprinted expression was dependent upon Smchd1, and (b) the region proximal to and including *Snrpn*, where genomic imprinting was maintained. This division of the cluster agrees with a previous model for the control of imprinting at the locus where genes from group (a) were controlled by DNA methylation and those from group (b) were regulated by the paternally expressed RNA transcript that initiated from the ICR [68]. To our knowledge, no other animal model has disassociated imprinting of the genes distal to *Snrpn* from the remainder of the cluster. The *Snrpn* cluster produces a poorly understood set of ncRNA transcript variants that are implicated in directing the imprinted expression of other genes within the cluster. Usually, these are expressed from the paternal allele and include the U-exon/Snrpn/Ube3a-ATS that has been proposed to control imprinted expression of *Ube3a* in the brain [69]. Recent reports have, however, identified yet other alternative *Snrpn* ncRNA transcripts emanating from upstream promoters and including upstream exons that are expressed from the maternal allele and required for silencing of genes on the maternal allele [70,71]. Our finding that Smchd1 is essential for some of the activities associated with the imprinting of the *Snrpn* cluster (that is, silencing the maternal allele of *Ndn*/*Magel2*/*Mkrn3* and *Peg12*/*Frat3*), but apparently not others (that is, silencing *Ube3a* on the paternal allele in the brain), adds further complexity to the regulation of this imprinted locus. Another new feature that has been identified in this study is the identification of a gene flanking *Ndn*, *Magel2*, and *Mkrn3*, which is likely to be imprinted and encodes a hypothetical novel tyrosine-rich region profile/EGF-like domain-containing protein.

Our SNP analysis of placental RNA-seq data also showed that the *Igf2r* imprinted gene cluster was disrupted in *Smchd1* mutants, with the *Slc22a3* gene showing loss of imprinting resulting in biallelic expression but imprinted *Igf2r* expression being unaffected. Imprinting of this cluster is governed by an ICR within exon 2 of *Igf2r*, which coincides with the transcriptional start site of the *Airn* ncRNA. It has recently been demonstrated that it is the transcriptional overlap of the *Airn* ncRNA and not the ncRNA transcript itself that induces the imprinting of *Igf2r*[72]. This is not the case for *Slc22a3*, whose imprinting is driven by the *Airn* ncRNA transcript itself, which targets G9a *in cis* to the allele of *Slc22a3* that is silenced [73]. Thus, we conclude that Smchd1 functions as part of the mechanism by which *Airn* ncRNA induces facultative heterochromatin of linked genes in the *Igf2r* imprinted gene cluster.

We specifically demonstrated that the differential DNA methylation marking the ICR associated with the *Snrpn* imprinted gene cluster was maintained. The sDMRs associated with the disrupted genes in this cluster were, however, almost completely unmethylated. Differential methylation of the ICR controlling the *Igf2r* imprinted cluster was also not altered in *Smchd1* mutants. The CpG island associated with *Slc22a3* gene does not appear to carry a differential methylation mark in the placenta [67] and was not assessed in this study.

It is possible that Smchd1 is involved in regulating the expression of further imprinted genes not identified in this study. A complete analysis would involve crossing the *Smchd1* mutant allele onto a suitable mouse strain carrying many SNPs in imprinted genes for determining allelic expression and analysis of suitable tissues where their expression is imprinted. If the imprinting of other genes involves Smchd1, we would predict that they would be situated within clusters of imprinted genes where the imprinting was regulated by the expression of an imprinted ncRNA similar to the situation for the *Snrpn* and *Igf2r* clusters. Our finding that in some *Smchd1*^MommeD1/MommeD1^ tissues the sDMR associated with *Cdkn1c* had altered levels of methylation may support this assertion, since regulation of the *Kcnq1* imprinted gene cluster involves the imprinted *Kcnq1ot1* ncRNA.

Our analysis has also highlighted altered expression of the clustered protocadherins in brain. These genes display an unusual form of random combinatorial monoallelic expression where each neuron expresses a unique combination of the protocadherin isoforms. This is proposed to confer individual neurons with a unique cell surface identity that may be important for neural circuit assembly [74]. The regulatory mechanism underlying the form of monoallelic expression displayed by protocadherins is poorly characterized but believed to involve the interaction of CTCF [75] with multiple enhancers and promoters [11,76]. We found that expression levels of some of the protocadherins, in particular that of *Pcdhα1*, was strongly deregulated in the *Smchd1* mutant brains (approximately 22-fold upregulated compared with wildtype). This is considerably more than the approximately 2-fold seen for the genes in the *Snrpn* imprinted gene cluster showing loss of imprinting, but this is possibly due to the unique form of random combinatorial monoallelic expression displayed by the clustered protocadherins, where *Pcdhα* chooses between 12 different alternative first exons to be utilized in each cell.

The exact molecular mechanism by which the Smchd1 protein functions in these roles remains unknown. *Smchd1* was named because the predicted encoded protein contains the hinge domain characteristic of SMC proteins [77]. The SMC proteins usually act as heterodimers (that is, SMC1 and 3 in cohesin, SMC2 and 4 in condensin, SMC5 and 6 in an unnamed complex involved in DNA repair) that interact through their hinge domains. The hinge domain of SMC proteins is also where the protein folds back on itself to bring together two nucleotide binding domains lying at the amino- and carboxy-terminuses of SMC proteins to form an ATPase domain, which hydrolyses ATP to provide the energy needed to manipulate chromosome-size molecules. The predicted Smchd1 protein also contains an ATPase domain, but it varies from the ABC-type ATPase contained in the SMC proteins and is similar to the ATPase domain from the GHKL phosphotransferase superfamily of type II DNA topoisomerases, Hsp90, and MutL, and bacterial and mitochondrial protein kinases [78]. From this, one would predict that Smchd1 might function as part of a protein complex that manipulates chromatin ultrastructure in an ATP-dependent manner. It is possible that Smchd1 is involved in physically sequestering the chromosomal region containing the allele being silenced to a nuclear territory that is remote from the active allele and where it can be stably maintained in its silent state. Such a function is consistent with the essential role we have previously shown for Smchd1 in X inactivation and with our current findings in the *Snrpn* and *Igf2r* imprinted gene clusters, and the clustered protocadherins.

## Conclusions

This work extends the involvement of Smchd1 from X inactivation to two other forms of monoallelic expression. Since the Killer cell lectin-like receptors (*Klra*), which are also subject to monoallelic expression, have previously also been reported to be deregulated in transformed MEFs and tumors that are mutant for Smchd1 [34], it will be interesting to determine whether Smchd1 can be implicated in other forms of monoallelic expression (that is, the olfactory receptors, T cell receptors). Moreover, *SMCHD1* mutation has recently been shown to contribute to an inherited muscular weakness syndrome, FSHD2, which requires digenic inheritance of a heterozygous *SMCHD1* mutation and a ‘permissive’ D4Z4 microsatellite array haplotype [79]. Heterozygous mutation of *SMCHD1* results in hypomethylation of the D4Z4 microsatellite array and, consequently, the DUX4 retrogene embedded in the array becomes expressed in skeletal muscle tissue in a variegated manner. A seemingly distinct role for Smchd1 has also been suggested with the identification *GMI1* as the likely *Arabidopsis* ortholog of *Smchd1*[80]. *GMI1* encodes a γ-radiation inducible protein that is proposed to be involved in homologous recombination. If Smchd1 also proves to function in this role in mammals, its diversity of function may rival that of the SMC proteins.

## Methods

### Mice and genotyping

All experimental animals were treated in accordance with the Australian Government National Health and Medical Research Council guidelines for the care of experimental animals and the work was approved by the animal ethics committees of the Queensland Institute of Medical Research and the Walter and Eliza Hall Institute. Mice carrying the mutant *Smchd1*^MommeD1^ allele were genotyped by allelic discrimination using a custom Taqman assay [31] (primers and Taqman probes are listed in Additional file 13). Mice carrying the ubiquitously expressed GFP transgene (UBI-GFP) were genotyped by observing GFP fluorescence.

### Embryos and cell cultures

All embryos were produced by natural matings between heterozygous (*Smchd1*^MommeD1/+^) males and females to produce wildtype, heterozygous, and homozygous *Smchd1* mutant embryos. Embryos for microarray analysis were dissected at E9.5, with the yolk sacs of each embryo used for genotyping. Embryos for generation of MEFs were dissected at E14.5 and a small section of the tail was removed for *Smchd1* genotyping. The remaining carcass of each embryo was individually treated for preparation of MEFs [81].

### Microarray analysis

Total RNA was extracted from *Smchd1*^MommeD1/MommeD1^ and *Smchd1*^+/+^ E9.5 embryos with RNA yields quantified using a Nanodrop ND-1000 spectrophotometer (Thermo Fisher Scientific Australia, Scoresby, Vic, Australia) and RNA integrity assessed using a Bioanalyzer (Agilent 2100, Agilent Laboratories, Santa Clara, CA, USA). Only samples with an RNA integrity number [82] greater than eight were used.

Illumina Mouse Ref-8 Expression BeadChips, v2.0 were hybridized with biotinylated cRNA prepared using Illumina TotalPrep RNA Amplification Kits (Applied Biosystems, Carlsbad, CA, USA). Probe annotation was enhanced using the Illumina BeadChip Probe Reannotation datasets [35]. Microarray data was analyzed in R using the Beadarray and Limma Bioconductor packages [83-85]. Gene set testing was performed separately for up- and downregulated genes on autosomal genes only, using DAVID [86].

### RNA-seq analysis

Total RNA was prepared from E9.5 embryos or the embryonic portion of placentas from F1 (C57Bl/6 J sire cross FVB/n dam) E14.5 male embryos dissected with the aid of a paternally inherited ubiquitin-GFP transgene [66]. RNA yields and integrity were assessed as for the microarrays. RNA-seq was performed by the Australian Genome Research Facility. The sequence reads were mapped to MM10 using TopHat 2 and expression differences between genotypes analyzed by Cufflinks/Cuffdiff v 2.0.2. For allele-specific expression, SNPs were identified in the RNA-seq sequence reads and allelic expression ratios quantified bioinformatically: SNPs were called in the RNA-seq data using the Samtools/BCFtools programs mpileup, bcftools and vcfutils, and mapped to the mouse RefSeq genes. For each called SNP and each sample, the ratio of reads supporting either the variant or reference was calculated. Significantly different allelic expression was identified by testing whether the means of these ratios were different in the *Smchd1*^+/+^ or *Smchd1*^MommeD1/MommeD1^ male embryos (using a two-tailed *t* test).

To confirm allelic bias, the placentas from reciprocal cross F1 mice (FVB/n sire cross C57Bl/6 J dam) were dissected and total RNA purified. RT-PCR was used to amplify the specific portions of transcripts containing the SNPs to be analyzed and PCR product used to generate barcoded libraries, which were pooled for Ion Torrent sequencing (Life Technologies, Grand Island, NY, USA). Allelic expression ratios were then determined from the mapped reads.

### qPCR and qRT-PCR

Quantitative PCR (qPCR and qRT-PCR) was performed using Platinum SYBR Green qPCR SuperMix-UDG (Life Technologies, Grand Island, NY, USA). Primers were designed using Primer3 and levels of specific PCR amplicons determined relative to a standard curve. For qRT-PCR, first-strand cDNA synthesis was primed either by oligo dT or by a cocktail of specific reverse primers. The sequences of all primers used are listed in Additional file 13.

### MeDIP analysis

MeDIP was performed using a protocol adapted from a published method [87]. Genomic DNA was sheared by sonication (Bioruptor, Diagenode, Liège, Belgium) to between 300 and 500 bp. DNA (1 μg) was then denatured and MeDIP performed with 1 μg of anti-5MeC monoclonal antibody clone 33D3 (ab10805, Abcam, Cambridge, UK) plus 0.5 mg of Dynabeads Protein G (Life Technologies, Grand Island, NY, USA). MeDIP material was purified by magnetic separation, phenol extraction, and precipitation. The recovery of specific genomic regions was measured by qPCR relative to *Rhox6*/*9*, as we had previously shown that CpG methylation at these loci was not affected by Smchd1 loss [31].

### ChIP analysis

ChIP for H3K4me2 was performed using an adaptation of a published protocol [88]. Chromatin was prepared from isolated nuclei of formaldehyde cross-linked MEFs and sonically sheared to between 200 and 500 bp. Sheared chromatin was immunoprecipitated with anti-H3K4me2 Ab (ab11946, Abcam, Cambridge, UK) plus 0.5 mg of Dynabeads ProteinG (Life Technologies, Grand Island, NY, USA). ChIP material was purified, as for MeDIP, and recovery of specific genomic regions in the ChIP samples was measured by qPCR relative to input DNA.

### Bisulfite sequencing

Nested primers for amplification of bisulfite converted DNA (Additional file 13) were designed using MethPrimer [89] and the sequencing data analyzed using BiQ analyzer [90]. Genomic DNA (1 μg) was bisulfite treated using the Qiagen EpiTect Bisulfite Kit (Qiagen, Hilden, Germany). After PCR, the product was purified (Qiagen PCR cleanup kit), cloned into pGemT (Promega, Madison, WI, USA) and colonies picked for PCR amplification with SP6 and T7 primers. The PCR product was sequenced with either SP6 or T7 primer.

### RNA-FISH

RNA-FISH was carried out as previously described [91] on *Smchd1*^+/+^ and *Smchd1*^MommeD1/MommeD1^ MEFs grown on gelatin-coated glass coverslips fixed with 3% paraformaldehyde. The probe for *Snrpn* was a BAC (RP23-97I5, RPCI-23 Female (C57BL/6 J) Mouse BAC Library), while the probes for *Ndn* and *Magel2* consisted of most of each gene PCR amplified from mouse genomic DNA (Additional file 12) and cloned into pGemT. The probes were labeled with Orange or Green dUTP (Abbott Molecular, Abbott Park, Illinois, USA) by nick translation and cell nuclei were counterstained with DAPI (1 μg/ml) before visualization on Zeiss Axioplan epifluorescence microscope equipped with a SPOT RT3 CCD camera. For quantification, 100 randomly selected nuclei were scored for the signal from each gene (that is, *Snrpn* and *Ndn* or *Magel2*).

### Data access

Microarray and RNA-seq data reported in this study have been submitted to the NCBI Gene Expression Omnibus under the accession numbers GSE44958 and GSE44669, respectively.

## Abbreviations

ChIP: Chromatin immunoprecipitation; ENU: N-ethyl-N-nitrosourea; FISH: Fluorescent *in situ* hybridization; GFP: Green fluorescent protein; H3K4me2: H3 dimethylated at lysine 4; H3K4me3: H3 trimethylated at lysine 4; H3K9me3: H3 trimethylated at lysine 9; H3K27me3: H3 trimethylated at lysine 27; ICR: Imprint control region; MeDIP: Methylated DNA immunoprecipitation; MEF: Mouse embryonic fibroblast; ncRNA: Noncoding RNA; NHMRC: National Health and Medical Research Council; qPCR: Quantitative PCR; qRT-PCR: Quantitative RT-PCR; sDMR: Secondary (or somatic) differentially methylated region; SNP: Single nucleotide polymorphism.

## Competing interests

The authors declare that they have no competing interests.

## Authors’ contributions

AWM carried out the molecular genetic studies, analyzed the data, and helped draft the manuscript. ZP helped in the molecular genetic studies and maintained the animals. MP helped collect embryonic tissue. IDT, MS, JD, and DC helped with the molecular genetic studies. AS, JJE, PM, and LK participated in next-generation sequencing data analysis. MJW analyzed the microarray data. MEB helped collect embryonic tissue, participated in the design of the study, and helped to draft the manuscript. GFK conceived the study, participated in its design and execution, and helped to draft the manuscript. All authors read and approved the final manuscript.

## Supplementary Material

Additional file 1 **Table of differentially expressed genes/probes from the microarray analysis of female E9.5 *Smchd1***^**MommeD1/MommeD1 **^**versus *Smchd1***^**+/+**^** embryos.** Click here for file

Additional file 2 **Table of differentially expressed genes/probes from the microarray analysis of male E9.5 *Smchd1***^**MommeD1/MommeD1 **^**versus *Smchd1***^**+/+ **^**embryos.** Click here for file

Additional file 3 **Gene set enrichment analysis of the autosomal gene classes altered in female E9.5 *Smchd1***^**MommeD1/MommeD1 **^**versus *Smchd1***^**+/+ **^**embryos.** Click here for file

Additional file 4 **Many X-linked genes may be upregulated, owing to X inactivation failure resulting from *Smchd1*-loss.** The mean log2 fold change of all expressed autosomal and X-linked genes (*A* value > 7.0) in *Smchd1*^MommeD1/MommeD1^ female embryos compared with *Smchd1*^+/+^ embryos is plotted. Click here for file

Additional file 5 **Confirmation of differential expression identified in microarrays.** Expression levels of the four most differentially expressed genes in the microarrays were quantified using qRT-PCR. RNA was derived from male and female *Smchd1*^+/+^ and *Smchd1*^MommeD1/MommeD1^ E9.5 embryos (samples included the four samples used for the microarrays but with two to four independent additional samples added, depending on genotype). The synthesis of first-strand cDNA was primed with oligo dT. In each case, the qRT-PCR signal was normalized relative to that of *Rala* and plotted relative to the corresponding *Smchd1*^+/+^ sample. The genotype, sex, and number of replicates are indicated in each case. Statistical analysis was performed using the *t* test. ** *P* < 0.01 and * *P* < 0.05 compared with wildtype. Error bars indicate standard error. Click here for file

Additional file 6 **Cuffdiff gene expression analysis of RNA-seq data comparing male E9.5 *Smchd1***^**MommeD1/MommeD1 **^**versus *Smchd1***^**+/+ **^**embryos.** Click here for file

Additional file 7 **RNA-seq reads (100 bp, single end sequencing of nondirectional RNA-seq libraries) identifying a gene flanking *Ndn***, ***Magel2 *and *Mkrn3.*** A screen shot from the UCSC Genome Browser showing RNA-seq reads from *Smchd1*^+/+^ and *Smchd1*^MommeD1/MommeD1^ male E9.5 embryos mapped to the genome in the region of *Ndn*, *Magel2* and *Mkrn3*. Only the RNA-seq reads that map to the region corresponding to the mouse mRNA corresponding to [AK142799, Genbank] are shown. RNA-seq reads mapping to the (+) strand are colored blue and those mapping to the (−) strand are colored red. RNA-seq reads that overlap exons are joined by a horizontal line. Click here for file

Additional file 8 **Validation of MeDIP results for MEFs using bisulfite sequencing.** DNA from several different MEF lines derived from individual E14.5 male *Smchd1*^+/+^ (left-hand panel) and *Smchd1*^MommeD1/MommeD1^ (right-hand panel) embryos was bisulfite treated and amplified with nested primers designed to amplify the ICR or sDMR of the imprinted genes being studied. Amplified product was cloned and sequenced to reveal the methylation status of CpG dinucleotides within the region of interest. For the *Snrpn* ICR, two different amplicons were analyzed (that is, *Snrpn* ICR region 1 and 2), while for the sDMR of *Ndn*, *Magel2*, and *Peg12*/*Frat3*, a single amplicon was designed at the edge of the CpG island that included as many as possible CpG dinucleotides while still giving reasonable amplification. In each case, no more than five individual clones were sequenced for each amplification or line of MEFs (groups from the same line of MEFs are joined by a line connecting the group) and only those clones with unique sequence were included. Methylated CpGs are indicated by the filled circles and unmethylated CpGs by open circles. Click here for file

Additional file 9 **MeDIP analysis of ICRs and sDMRs for several imprinted gene clusters in (A) male and female E9.5 embryos, and (B) MEFs derived from male E14.5 embryos.** The genotype, sex, and number of replicates are indicated in each case. Statistical analysis was performed using the *t* test. * *P* < 0.05 compared with wildtype. Error bars indicate standard error. Click here for file

Additional file 10 **Cuffdiff gene expression analysis of RNA-seq data comparing the placenta from male E14.5 *Smchd1***^**MommeD1/MommeD1 **^**against *Smchd1***^**+/+ **^**F1 (C57Bl6/J × FVB/n) embryos.** Click here for file

Additional file 11 **SNP analysis of RNA-seq data comparing the placenta from male E14.5 *Smchd1***^**MommeD1/MommeD1 **^**against *Smchd1***^**+/+ **^**F1 (C57Bl6/J × FVB/n) embryos.** Click here for file

Additional file 12 **Bisulfite analysis of the *Igf2r *ICR in placental tissue.** DNA from the embryonic portion of the placenta derived from individual E14.5 male *Smchd1*^+/+^ (left-hand panel) and *Smchd1*^MommeD1/MommeD1^ (right-hand panel) embryos was bisulfite treated and amplified with nested primers designed to amplify the ICR of the *Igf2r* imprinted gene cluster. Amplified product was cloned and sequenced to reveal the methylation status of CpG dinucleotides. Only those clones with unique sequences were included. Clones derived from the same placenta are joined by a line connecting the group and only those clones with unique sequence were included. Methylated CpGs are indicated by the filled circles and unmethylated CpGs by open circles. Click here for file

Additional file 13 The complete list of PCR primers used. Click here for file
