# Supplementary figures and images for: Smchd1 regulates a subset of autosomal genes subject to monoallelic expression in addition to being critical for X inactivation
Source: Epigenetics Chromatin. 2013 Jul 2;6:19. doi: 10.1186/1756-8935-6-19 (PMC3707822; doi:10.1186/1756-8935-6-19)

# Change in gene expression in MommeD1 Females

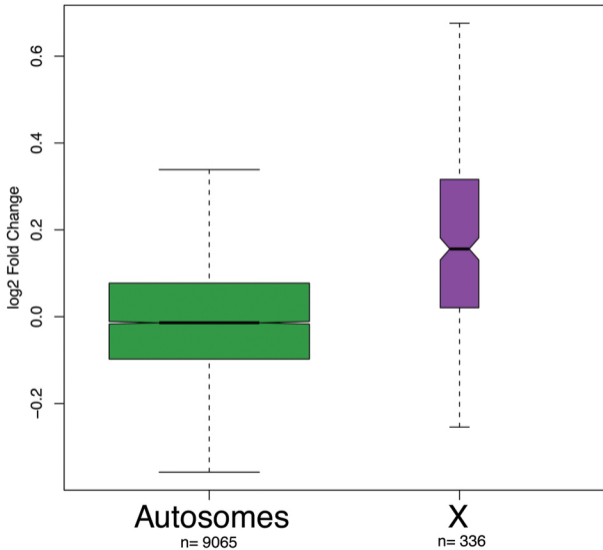

Supplement: Additional file 4 — Many X-linked genes may be upregulated, owing to X inactivation failure resulting from Smchd1-loss. The mean log2 fold change of all expressed autosomal and X-linked genes (A value > 7.0) in Smchd1MommeD1/MommeD1 female embryos compared with Smchd1+/+ embryos is plotted. [file 1756-8935-6-19-S4.pdf]

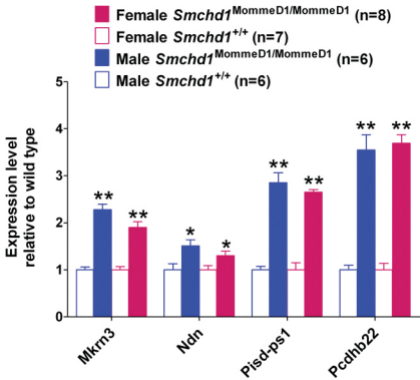

Supplement: Additional file 5 — Confirmation of differential expression identified in microarrays. Expression levels of the four most differentially expressed genes in the microarrays were quantified using qRT-PCR. RNA was derived from male and female Smchd1+/+ and Smchd1MommeD1/MommeD1 E9.5 embryos (samples included the four samples used for the microarrays but with two to four independent additional samples added, depending on genotype). The synthesis of first-strand cDNA was primed with oligo dT. In each case, the qRT-PCR signal was normalized relative to that of Rala and plotted relative to the corresponding Smchd1+/+ sample. The genotype, sex, and number of replicates are indicated in each case. Statistical analysis was performed using the t test. ** P < 0.01 and * P < 0.05 compared with wildtype. Error bars indicate standard error. [file 1756-8935-6-19-S5.pdf]

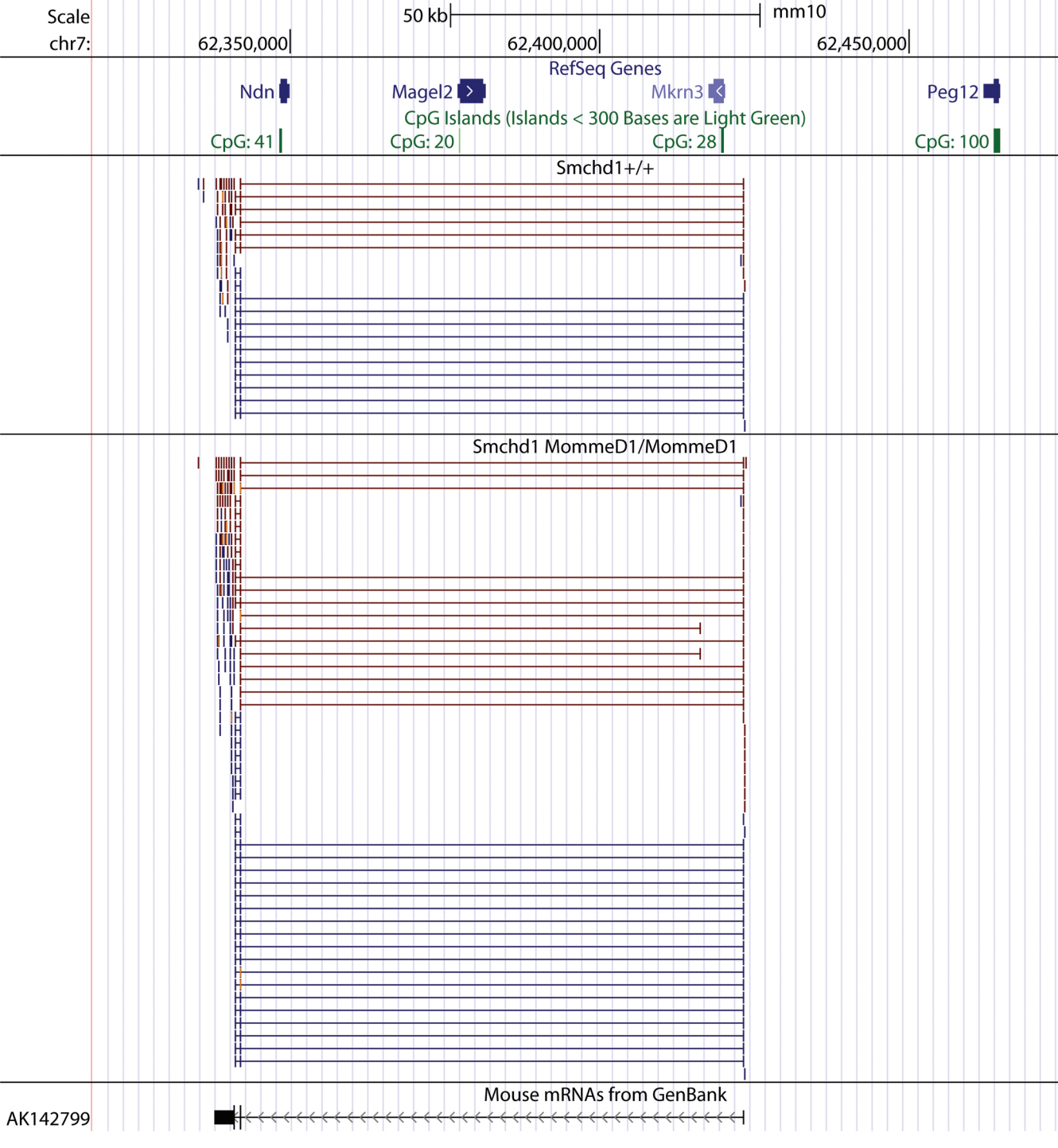

Supplement: Additional file 7 — RNA-seq reads (100 bp, single end sequencing of nondirectional RNA-seq libraries) identifying a gene flanking Ndn, Magel2 and Mkrn3. A screen shot from the UCSC Genome Browser showing RNA-seq reads from Smchd1+/+ and Smchd1MommeD1/MommeD1 male E9.5 embryos mapped to the genome in the region of Ndn, Magel2 and Mkrn3. Only the RNA-seq reads that map to the region corresponding to the mouse mRNA corresponding to [AK142799, Genbank] are shown. RNA-seq reads mapping to the (+) strand are colored blue and those mapping to the (−) strand are colored red. RNA-seq reads that overlap exons are joined by a horizontal line. [file 1756-8935-6-19-S7.pdf]

**A**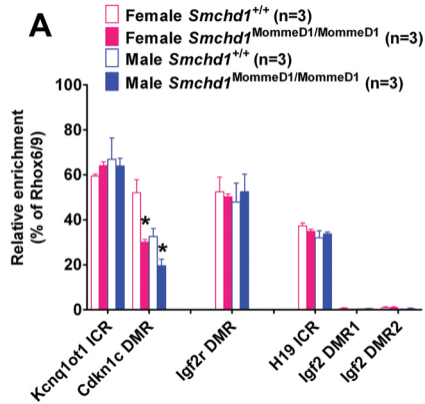**B**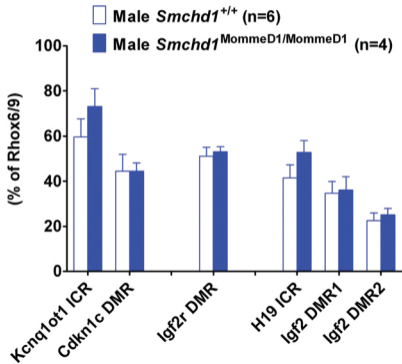

Supplement: Additional file 9 — MeDIP analysis of ICRs and sDMRs for several imprinted gene clusters in (A) male and female E9.5 embryos, and (B) MEFs derived from male E14.5 embryos. The genotype, sex, and number of replicates are indicated in each case. Statistical analysis was performed using the t test. * P < 0.05 compared with wildtype. Error bars indicate standard error. [file 1756-8935-6-19-S9.pdf]

# Smchd1<sup>+/+</sup> Placenta

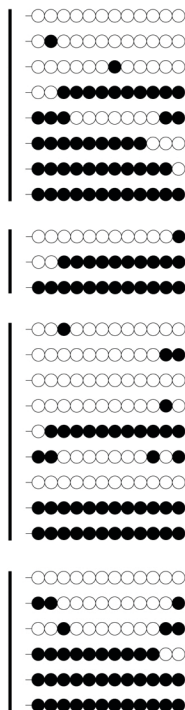

# Smchd1<sup>MommeD1/MommeD1</sup> Placenta

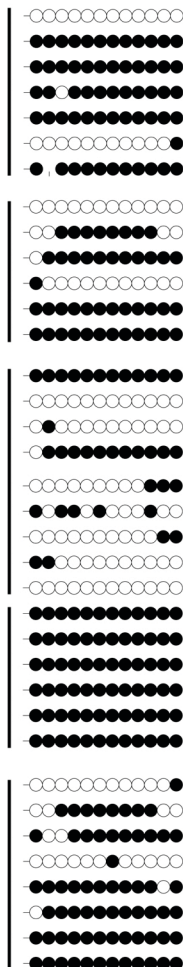

Supplement: Additional file 12 — Bisulfite analysis of the Igf2r ICR in placental tissue. DNA from the embryonic portion of the placenta derived from individual E14.5 male Smchd1+/+ (left-hand panel) and Smchd1MommeD1/MommeD1 (right-hand panel) embryos was bisulfite treated and amplified with nested primers designed to amplify the ICR of the Igf2r imprinted gene cluster. Amplified product was cloned and sequenced to reveal the methylation status of CpG dinucleotides. Only those clones with unique sequences were included. Clones derived from the same placenta are joined by a line connecting the group and only those clones with unique sequence were included. Methylated CpGs are indicated by the filled circles and unmethylated CpGs by open circles. [file 1756-8935-6-19-S12.pdf]
