# Supplementary material for: Smchd1 regulates a subset of autosomal genes subject to monoallelic expression in addition to being critical for X inactivation
Source: Epigenetics Chromatin. 2013 Jul 2;6:19. doi: 10.1186/1756-8935-6-19 (PMC3707822; doi:10.1186/1756-8935-6-19)
Supplement: Additional file 8 — Validation of MeDIP results for MEFs using bisulfite sequencing. DNA from several different MEF lines derived from individual E14.5 male Smchd1+/+ (left-hand panel) and Smchd1MommeD1/MommeD1 (right-hand panel) embryos was bisulfite treated and amplified with nested primers designed to amplify the ICR or sDMR of the imprinted genes being studied. Amplified product was cloned and sequenced to reveal the methylation status of CpG dinucleotides within the region of interest. For the Snrpn ICR, two different amplicons were analyzed (that is, Snrpn ICR region 1 and 2), while for the sDMR of Ndn, Magel2, and Peg12/Frat3, a single amplicon was designed at the edge of the CpG island that included as many as possible CpG dinucleotides while still giving reasonable amplification. In each case, no more than five individual clones were sequenced for each amplification or line of MEFs (groups from the same line of MEFs are joined by a line connecting the group) and only those clones with unique sequence were included. Methylated CpGs are indicated by the filled circles and unmethylated CpGs by open circles. [file 1756-8935-6-19-S8.pdf]

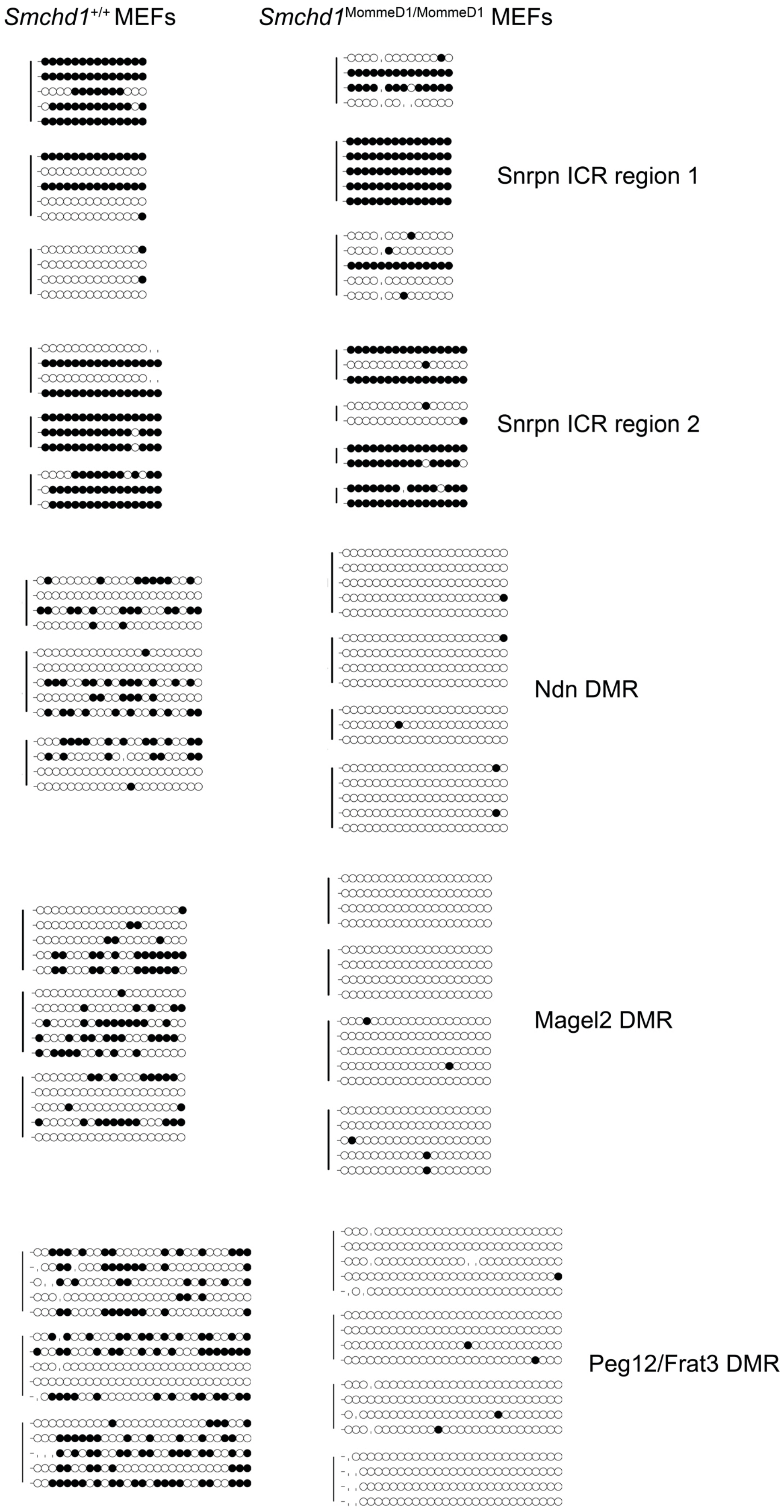

Smchd1+/+ MEFs
Smchd1MommeD1/MommeD1 MEFs
Snrpn ICR region 1
Snrpn ICR region 2
Ndn DMR
Magel2 DMR
Peg12/Frat3 DMR
